# Functions of the WNT Signaling Network in Shaping Host Responses to Infection

**DOI:** 10.3389/fimmu.2019.02521

**Published:** 2019-11-08

**Authors:** Johanna K. Ljungberg, Jessica C. Kling, Thao Thanh Tran, Antje Blumenthal

**Affiliations:** The University of Queensland Diamantina Institute, The University of Queensland, Brisbane, QLD, Australia

**Keywords:** WNT signaling, antigen presenting cells (APCS), infection, inflammation, anti-microbial defense

## Abstract

It is well-established that aberrant WNT expression and signaling is associated with developmental defects, malignant transformation and carcinogenesis. More recently, WNT ligands have emerged as integral components of host responses to infection but their functions in the context of immune responses are incompletely understood. Roles in the modulation of inflammatory cytokine production, host cell intrinsic innate defense mechanisms, as well as the bridging of innate and adaptive immunity have been described. To what degree WNT responses are defined by the nature of the invading pathogen or are specific for subsets of host cells is currently not well-understood. Here we provide an overview of WNT responses during infection with phylogenetically diverse pathogens and highlight functions of WNT ligands in the host defense against infection. Detailed understanding of how the WNT network orchestrates immune cell functions will not only improve our understanding of the fundamental principles underlying complex immune response, but also help identify therapeutic opportunities or potential risks associated with the pharmacological targeting of the WNT network, as currently pursued for novel therapeutics in cancer and bone disorders.

## The WNT Signaling Network

The WNT signaling network is a central regulator of embryonic development and tissue homeostasis. WNT proteins are phylogenetically highly conserved secreted, cysteine-rich glyco-lipoproteins (1). Nineteen individual WNT proteins have thus far been described in mammals (2). Best known functions of WNT proteins include regulation of cell cycle, cellular differentiation, cell motility, cellular polarity, and cell death (3). WNT proteins act as directional growth factors that orchestrate patterning, expansion and differentiation of tissues in the organized formation of body plans, and are central regulators of stem and progenitor cell development and maintenance both during embryogenesis and adult homeostasis (4, 5). Dysregulation of WNT signaling is implicated in a multitude of diseases, including cancer, fibrosis, bone density disorders, metabolic and neurodegenerative diseases (6).

**Figure 1:**
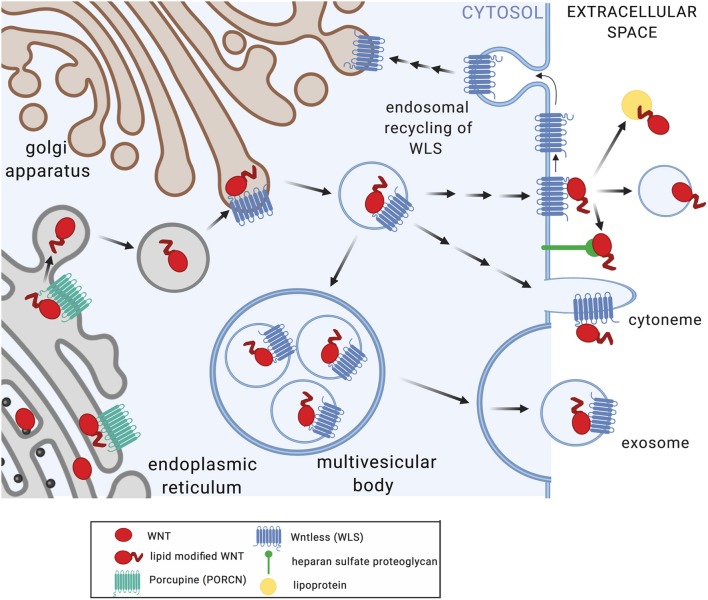
Posttranslational modification and secretion of WNT ligands. Upon translation, WNT proteins undergo acylation in the endoplasmic reticulum by Porcupine (PORCN), a modification required for WNT release (7), receptor interactions (8), and functions (7, 9). Wntless (WLS) facilitates transport of acylated WNT ligands to the plasma membrane and aids in WNT release (10–12). WNT secretion and extracellular transport appears to occur via multiple mechanisms including multi-vesicular bodies and exosomes, cytonemes, lipoproteins, and heparan sulfate proteoglycans (1). WLS protein is recycled via clathrin-mediated endocytosis (13). Figure created with Biorender.com.

WNT proteins are highly hydrophobic due to post-translational modification by palmitoleic and palmitic acid at conserved cysteine residues. This is afforded through action of the acyltransferase Porcupine (PORCN) in the endoplasmic reticulum (Figure 1). WNT acylation has been shown to be required for the release, receptor interactions, and functions of WNTs (1). The chaperone Wntless (WLS) facilitates transport of acylated WNT ligands to the plasma membrane and aids in WNT release (14). WNT proteins act as morphogens in a concentration-dependent manner through the formation of gradients within tissues. How the hydrophobic WNT ligands act at short distances as well as at longer ranges is incompletely understood. Multiple mechanisms that facilitate WNT transport beyond the boundaries of the producing cell have been described, including chaperones, lipoproteins, exosomes, and cytonemes (1). Macrophages infected by viruses or intracellular bacterial pathogens release exosomes and microvesicles that contain pathogen-derived components alongside host membrane proteins (e.g., MHC-I, MHC-II) and immune mediators (e.g., cytokines) that modulate functions of bystander cells (15–18). Observations of elevated WNT5A protein levels in serum of patients with severe sepsis (19) highlights the possibility that WNT proteins produced in response to infection may act not only locally but also systemically, and thereby shape immune cell differentiation and functions at distant sites.

**Figure 2:**
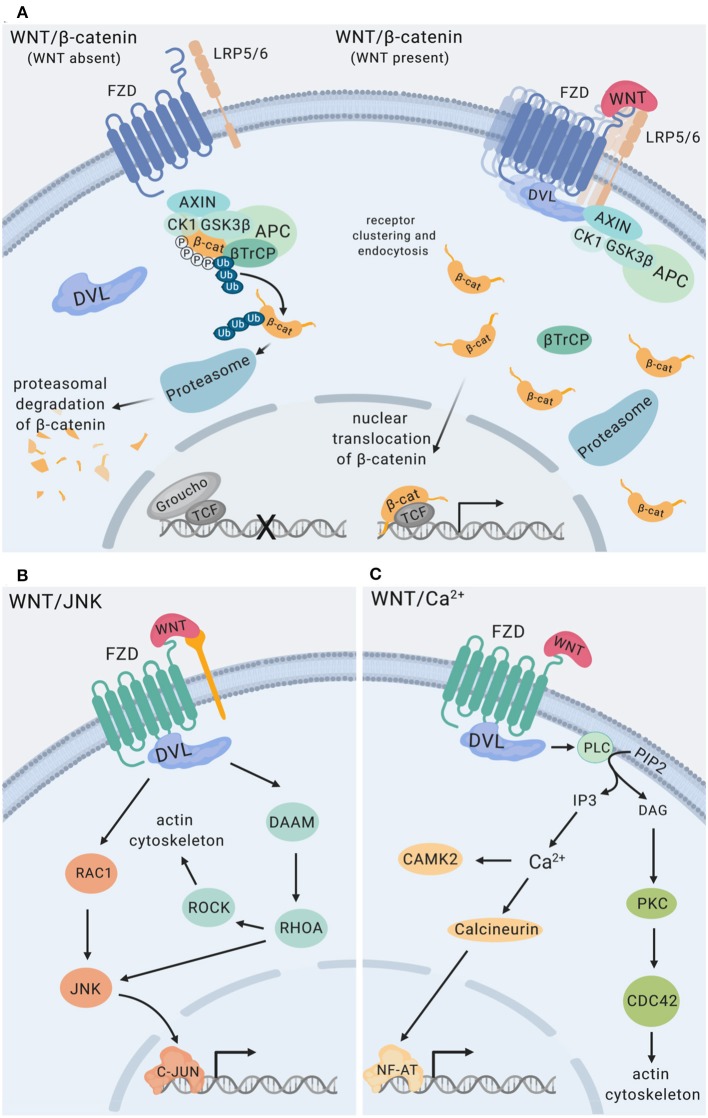
WNT signaling pathways. **(A)** WNT/β-catenin signaling. The destruction complex is comprised of APC, AXIN1, CK1, and GSK3β. Phosphorylation of β-catenin by CK1 and GSK3β within the destruction complex results in β-catenin ubiquitination mediated by β-TrCP resulting in proteasomal degradation of β-catenin (22, 23) The transcriptional repressor Groucho suppresses expression of genes controlled by TCF/LEF transcription factors. Binding of WNT ligands to Frizzled (FZD) receptors and LRP co-receptors promotes recruitment and clustering of DVL, forming signalosomes (21, 24), facilitating recruitment of the destruction complex and stabilization of cytoplasmic β-catenin. Nuclear translocation of β-catenin enables its functions as a transcriptional co-activator for transcription factors of the TCF/LEF family (3). **(B)** WNT/JNK signaling via FZD, alone or in conjunction with co-receptors (e.g., ROR RYK) activates the small GTPases RAC1 and RHOA engaging the actin cytoskeleton, as well as JNK MAP kinase activation (25–27). **(C)** WNT/Ca^2+^ signaling downstream of FZD receptors is mediated by phospholipase C (PLC) activation leading to enhanced levels of cytosolic Ca^2+^, resulting in calmodulin/calmodulin-dependent kinase II activation and NF-AT-regulated transcriptional responses (28), and engagement of the actin cytoskeleton. RYK has been implicated as a co-receptor for WNT/Ca^2+^ signaling. Figure created with Biorender.com.

WNT ligands initiate intracellular signaling by binding to cell surface-expressed WNT receptors and co-receptors, including Frizzled (FZD) 7-transmembrane domain receptors, low-density lipoprotein-related proteins (LRP5, LRP6), as well as receptor tyrosine kinases ROR and RYK (20). Cytoplasmic scaffolding proteins of the disheveled family (DVL) are central to initiating intracellular signaling downstream of FZD receptors (21). The functional outcome of WNT interactions with target cells is decided at the level of receptor engagement. Depending on the receptor context, WNT ligands activate distinct intracellular pathways, which can be grouped into β-catenin-dependent and β-catenin-independent signaling events (Figure 2). Individual modalities of β-catenin-dependent and β-catenin-independent WNT signaling have been reviewed in detail elsewhere (3, 5, 20). Briefly, β-catenin-dependent WNT signaling is mediated by cytoplasmic stabilization of β-catenin, which is controlled by the β-catenin destruction complex. The destruction complex is comprised of scaffolding proteins adenomatous polyposis coli (APC), axis inhibition protein (Axin), and the kinases casein kinase 1 (CK1) and glycogen synthase kinase 3β (GSK3β). In the absence of WNT ligand binding to FZD and LRP co-receptors, phosphorylation of β-catenin by CK1 and GSK3β within the destruction complex results in β-catenin ubiquitination by beta-transducin repeat-containing E3 ubiquitin protein ligase (βTrCP), fueling continuous degradation of β-catenin by the proteasome (Figure 2A). Binding of WNT ligands to FZD/LRP results in recruitment of DVL and the destruction complex, inhibiting GSK3β and CK1 activity and stabilization of cytoplasmic β-catenin. This enables nuclear translocation of β-catenin where it functions as transcriptional co-activator for transcription factors of the TCF/LEF family (Figure 2A). WNT/JNK- [described as planar cell polarity (PCP) pathway in *Drosophila*] and WNT/Ca^2+^-signaling are modes of β-catenin-independent WNT signaling. WNT/JNK signaling results in FZD/DVL-mediated activation of the small GTPases RAC1 and RHOA, directing cytoskeletal rearrangements, cell polarization and motility. Activation of JNK can drive c-Jun- and AP-1-controlled transcription (Figure 2B). WNT/Ca^2+^ signaling downstream of FZD receptors and DVL leads to phospholipase C (PLC) activation and enhanced levels of cytosolic Ca^2+^, which activates calmodulin/calmodulin-dependent kinase II and NFAT-regulated transcriptional responses (Figure 2C).

Tight regulation and precise targeting of WNT signaling is essential, as emphasized by the evolutionary investment in multiple layers and modes of WNT pathway modulation. WNT signaling is negatively regulated by secreted Frizzled-related proteins (sFRP) and WNT inhibitory factor 1 (WIF-1), which directly bind WNT proteins interfering with receptor interactions (3). The palmitoleoyl-protein carboxylesterase Notum was shown to facilitate serine de-palmitoleoylation of WNT ligands, thereby negatively regulating WNT functions (29). Members of the Dickkopf (DKK) and Sclerostin/SOST families, as well as the glycoprotein Dorsal Inhibitory Axon Guidance Protein (DRAXIN) interact with LRP5/6 and interfere with WNT binding (30–32). FZD receptor surface availability is regulated through the E3 ubiquitin ligases, Zinc and Ring Finger 3 (ZNRF3) and Ring Finger protein 43 (RFN43), which ubiquitinate FZD receptors destining them for proteasomal degradation (33). ZNFR3 and RFN43 serve as negative feedback regulators for WNT signaling, as they themselves are encoded by WNT target genes (5).

## WNT Responses to Infection

Early studies identified *WNT5A* as a highly responsive gene in human macrophages upon microbial encounter (19, 34). *WNT5A* has also been found to be highly expressed by tumor-associated macrophages (35), synoviocytes in rheumatoid arthritis (36), macrophages in atherosclerotic plaques (37), and adipose tissue-resident macrophages in obesity (38). This has directed initial attention toward elucidating immune functions of WNT5A. However, it is increasingly evident that the host response to infection encompasses differential expression of multiple WNT ligands, receptors and regulators (39–43). Thus, detailed understanding of how the concerted actions of WNT ligands and potentially concurrent WNT signaling events define host responses to infection is key to firmly establishing immune functions of the WNT signaling network.

### Bacterial Infections

#### Gram-Negative Bacteria

WNT responses to infection have been studies in the context of experimental infection with a limited number of Gram-negative bacterial pathogens (Table 1). WNT pathway activation and functions in the context of *Salmonella* infection have largely been focused on in a model of gastroenteritis in antibiotic-pretreated mice, as well as in epithelial cell lines *in vitro*. *Salmonella (S.) enterica* serovar Typhimurium infection of streptomycin-pretreated mice increased mRNA expression of *Wnt3, Wnt6, Wnt9a*, and protein expression of Wnt2 and Wnt11 in intestinal tissues (43, 50, 56). *In vitro* studies indicated that colonization of murine intestinal epithelial cells with *S*. Typhimurium induced elevated mRNA expression of *Wnt2* and *Wnt11* (also confirmed at protein level), *Fzd2, Fzd4, Fzd6, Fzd7, Fzd8, Fzd9*, with limited or no effects on the expression of other *Wnt* and *Fzd* genes (50, 56). Induction of Wnt2 and Wnt11 expression was attributed at least in part to *Salmonella* AvrA (50, 56), a bacterial effector that has been implicated in the regulation of β-catenin ubiquitination and stabilization (64–67). With an increasing understanding of the complex WNT response in *Salmonella* infection, future studies should explore WNT network activation in macrophages, innate immune cells that are important in the host control of *Salmonella* infection. Thus far, it has been noted that *Wnt5a* and *Fzd4* expression in *S*. Typhimurium-infected murine peritoneal macrophages was modestly increased, albeit the impact on the expression of other WNT signaling components was not explored in this study (44).

**Table 1:** Bacteria-induced WNT responses in experimental systems and patient samples.

	***Mycobacterium* sp**.	***S. aureus***	***S. pneumoniae***	***E. chaffeensis***	***E. coli***	***P. aeruginosa***	***S*. Typhimurium**	**LPS**
WNT1	↑ mRNA *Mtb* murine lung tissue (40) unaffected mRNA *M. bovis* BCG mPM (44)	↓ mRNA *mom-2 C. elegans* IEC (45) unaffected mRNA mPM (44) unaffected *L. vannamei* HP, LO, HT (46)	–	n.d. mRNA THP-1 (42)	unaffected mRNA mPM (44)	–	↓protein HCT116 (47)	↑ protein THP-1 (48) ↑ mRNA mBMDM (40) ↓ mRNA fetal ovine lungs (49) n.d. mRNA murine spleen (39)
WNT2	↓ mRNA *Mtb* murine lung tissue (40) unaffected mRNA *M. bovis* BCG mPM (44)	↑ mRNA *cwn-2 C. elegans* IEC (45) unaffected mRNA mPM (44) unaffected *L. vannamei* HP, LO, HT (46)	–	n.d. mRNA THP-1 (42)	↑ mRNA and protein CMT93 (50) unaffected mRNA mPM (44)	–	↑ mRNA and protein IEC-18 (50)	n.d. mRNA murine spleen (39) ↓ mRNA fetal ovine lungs (49)
WNT2B	↓ mRNA *Mtb* murine lung tissue (40) unaffected mRNA *M. bovis* BCG mPM (44)	unaffected mRNA mPM (44)	↓ mRNA murine *S. pneumoniae* Δpep27 lung tissue (51)	unaffected mRNA THP-1 (42)	unaffected mRNA mPM (44)	–	unaffected mRNA IEC-18 (50)	↑ mRNA mBMDM (40) n.d. mRNA murine spleen (39)
WNT3	unaffected mRNA *Mtb* murine lung tissue (40)	–	↓ mRNA murine *S. pneumoniae* Δpep27 lung tissue (51)	unaffected mRNA THP-1 (42)	unaffected mRNA mPM (44)	–	↑ mRNA mIEC (43)	↑ mRNA hMDM (52) unaffected mRNA murine spleen (39)
WNT3A	↓ mRNA *Mtb* murine lung tissue (40) unaffected mRNA *M. bovis* BCG mPM (44)	unaffected mRNA mPM (44)	–	n.d. mRNA THP-1 (42)	unaffected mRNA mPM (44)	–	unaffected mRNA IEC-18 (50)	n.d. mRNA mBMDM (40) ↑ mRNA BEAS-2B (53) n.d. mRNA murine spleen (39)
WNT4	↓ mRNA *Mtb* murine lung tissue (40) ↓ mRNA *M. marinum* infected zebrafish (54) unaffected mRNA *M. bovis* BCG mPM (44)	↑ mRNA *D. melanogaster* (55) unaffected mRNA mPM (44) unaffected *L. vannamei* HP, LO, HT (46)	↑ mRNA murine *S. pneumoniae* Δpep27 lung tissue (51)	unaffected mRNA THP-1 (42)	unaffected mRNA mPM (44)	–	unaffected mRNA IEC-18 (56)	↓ mRNA fetal ovine lungs (49) unaffected mRNA murine spleen (39)
WNT5A	↑ mRNA *Mtb* hMDM (34) ↓ mRNA *Mtb* murine lung tissue (40) ↑ mRNA *Mtb* hPBMC and *M. bovis* (BCG) mPM (44) ↑ mRNA *M. marinum* zebrafish (54) WNT5A expressing macrophages in human tuberculosis granulomas (34)	↑ mRNA mPM (44) ↑ mRNA (*LvWnt5*) *L. vannamei* HP, LO, HT (46)	↓ protein RAW264.7 (57)	n.d. mRNA THP-1 (42)	↓ mRNA mPM (44) ↓ mRNA murine urothelium (58) ↑ mRNA human urothelium (59)	↓ protein RAW264.7 (57)	↑ mRNA mPM (44) unaffected mRNA IEC-18 (56)	↑ mRNA hMDM (34) ↑ mRNA BEAS-2B (53) ↑ mRNA hPBMC and hBMDM during sepsis (19) ↑ mRNA THP-1 (60) ↑ mRNA primary human monocytes (61) ↑ mRNA RAW264.7 (37) unaffected mRNA murine spleen (39) unaffected mBMDM (40)
WNT5B	↓ mRNA *Mtb* murine lung tissue (40) unaffected mRNA *M. bovis* BCG mPM (44)	unaffected mRNA mPM (44) ↑ mRNA (*LvWnt5*) *L. vannamei* HP, LO, HT (46)	↑ mRNA murine *S. pneumoniae* Δpep27 lung tissue (51)	↓ mRNA THP-1 (42)	unaffected mRNA mPM (44)	–	unaffected mRNA IEC-18 (56)	↑ mRNA mBMDM (40) ↑ mRNA murine spleen (39)
WNT6	↑ mRNA *Mtb* mBMDM (40) unaffected mRNA *M. bovis* BCG mPM (44)	unaffected mRNA mPM (44) ↑ mRNA *L. vannamei* HP, unaffected LO, HT (46)	↓ mRNA murine *S. pneumoniae* Δpep27 lung tissue (51)	↑ mRNA THP-1 (42)	unaffected mRNA mPM (44)	–	↑ mRNA mIEC (43)	↓ mRNA murine spleen (39) ↑ mRNA mBMDM (40)
WNT7A	↓*Mtb* murine lung tissue (40) unaffected mRNA *M. bovis* BCG mPM (44)	unaffected mRNA mPM (44) unaffected mRNA (*LvWnt7*) *L. vannamei* HP, LO, HT (46)	↑ mRNA murine *S. pneumoniae* Δpep27 lung tissue (51)	n.d. mRNA THP-1 (42)	unaffected mRNA mPM (44)	–	–	n.d. mRNA mBMDM (40) n.d. mRNA murine spleen (39)
WNT7B	unaffected mRNA *Mtb* murine lung tissue (40) unaffected mRNA *M. bovis* BCG mPM (44)	unaffected mRNA mPM (44) unaffected mRNA (*LvWnt7*) *L. vannamei* HP, LO, HT (46)	↑ mRNA murine *S. pneumoniae* Δpep27 lung tissue (51)	↓ mRNA THP-1 (42)	unaffected mRNA mPM (44)	–	–	n.d. mRNA mBMDM (40) ↑ mRNA fetal ovine lungs (49) n.d. mRNA murine spleen (39)
WNT8A	↓ mRNA *Mtb* murine lung tissue (40) unaffected mRNA *M. bovis* BCG mPM (44)	unaffected mRNA mPM (44) unaffected mRNA (*LvWnt8*) *L. vannamei* HP, LO, HT (46)	–	unaffected THP-1 (42)	unaffected mRNA mPM (44)	–	–	n.d. mRNA mBMDM (40) n.d. mRNA murine spleen (39)
WNT8B	↓ mRNA *Mtb* murine lung tissue (40) unaffected mRNA *M. bovis* BCG mPM (44)	unaffected mRNA mPM (44) unaffected mRNA (*LvWnt8*) *L. vannamei* HP, LO, HT (46)	–	n.d. mRNA THP-1 (42)	unaffected mRNA mPM (44)	–	–	n.d. mRNA murine spleen (39)
WNT9A	↓ mRNA *Mtb* murine lung tissue (40) unaffected mRNA *M. bovis* BCG mPM (44)	unaffected mRNA mPM (44) ↑ mRNA (*LvWnt9*) *L. vannamei* lymphoid organ (46) unaffected mRNA (*LvWnt9*) *L. vannamei* HP, LO, HT (46)	↓ mRNA murine *S. pneumoniae* Δpep27 lung tissue (51)	unaffected mRNA THP-1 (42)	unaffected mRNA mPM (44)	–	↑ mRNA mIEC (43)	n.d. mRNA murine spleen (39)
WNT9B	↓ mRNA *Mtb* murine lung tissue (40) unaffected mRNA *M. bovis* BCG mPM (44)	unaffected mRNA mPM (44) ↑ mRNA (*LvWnt9*) *L. vannamei* lymphoid organ (46) unaffected mRNA (*LvWnt9*) *L. vannamei* HP, LO, HT (46)	↓ mRNA murine *S. pneumoniae* Δpep27 lung tissue (51)	n.d. mRNA THP-1 (42)	unaffected mRNA mPM (44)	–	–	n.d. mRNA murine spleen (39)
WNT10A	↑ mRNA *Mtb* murine lung tissue (40) ↓ mRNA *M. marinum* zebrafish (54)	unaffected mRNA (*LvWnt10*) *L. vannamei* HP, LO, HT (46)	–	↑ mRNA THP-1 (42)	unaffected mRNA mPM (44)	–	–	↑ mRNA murine spleen (39) unaffected mRNA mBMDM (40)
WNT10B	↓ mRNA *Mtb* murine lung tissue (40) unaffected mRNA *M. bovis* BCG mPM (44)	unaffected mRNA mPM (44) unaffected mRNA (*LvWnt10*) *L. vannamei* HP, LO, HT (46)	–	n.d. mRNA THP-1 (42)	unaffected mRNA mPM (44)	–	unaffected mRNA IEC-18 (50)	↑ mRNA murine spleen (39) ↑ mRNA mBMDM (40)
WNT11	unaffected mRNA *M. bovis* BCG mPM (44)	unaffected mRNA mPM (44) unaffected mRNA *L. vannamei* HP, LO, HT (46)	–	unaffected mRNA THP-1 (42)	unaffected mRNA mPM (44)	–	↑ mRNA and protein IEC-18 (56)	unaffected mBMDM (40) ↑ mRNA murine spleen (39)
WNT16	↓ mRNA *Mtb* murine lung tissue (40) ↓ mRNA *M. marinum* zebrafish (54)	unaffected mRNA *L. vannamei* HP, LO, HT (46)	–	n.d. mRNA THP-1 (42)	unaffected mRNA mPM (44)	–	–	unaffected mRNA murine spleen (39)
Effects not specific to individual WNT proteins	↑*Fzd1 Mtb* murine lung tissue and mBMDM (41) ↑*Fzd4, Lrp5*, β-catenin stabilization through GSK3 phosphorylation in *M. bovis* BCG mPM (44) ↓*Axin2, Dvl3, Fzd8,9,10* ↑*Dvl2 M. marinum* zebrafish (54)	↓*mom-5, mig-1* (FZD homologues) *C. elegans* IEC (45) ↑*Fzd4* m. PM (44) ↑ mRNA *LvWntA L. vannamei* HP, LO (46)	↓β-catenin activity murine lung tissue (62)	↓*FZD7* ↑*FZD5, 9* ↓*DKK3* ↑*sFRP2* THP-1 (42)	-	↓*Ctnnb1* RAW264.7 (63)	β-catenin degradation IEC-18 (64) ↑*Fzd4, 6, 9, Fzd3, 10* unaffected IEC-18 (50) ↑*FZD2, 7, 8 FZD5* unaffected HCT116 (56) ↑*Fzd4* mPM (44)	↑*Fzd1, 5, Wisp1*, β*-catenin* ↓*Fzd7, 8 Fzd3, 6, 9*, and *10* unaffected murine spleen (39) ↓*Ctnnb1* fetal ovine lungs (49) ↑ LRP6 phosphorylation BEAS-2B (53) ↑*DVL3* hMDM (34)

*mRNA or protein expression of WNT ligands and signaling components in response to infection or LPS exposure. ↑, upregulation; ↓, downregulation; –, indicative of no data; n.d., not detectable; protein, protein expression; mRNA, mRNA expression; m, murine; h, human; PM, peritoneal macrophages; BMDM, bone marrow-derived macrophages; IEC, intestinal epithelial cells; MDM, monocyte-derived macrophages; PBMC, peripheral blood mononuclear cells; SMC, spleen mononuclear cells; HP, hepatopancreas; LO, lymphoid organs; HT, hemocytes; Mtb, Mycobacterium tuberculosis*.

*Ehrlichia (E.) chaffeensis* infection of human THP-1 macrophage-like cells transiently increased mRNA expression of *WNT6, WNT10A, FZD5*, and *FZD9*, while decreasing expression of *WNT5B, WNT7B*, and *FZD7*, as determined by pathway-specific qPCR arrays (42). Expression of WNT regulators such as *DKK3* and *sFRP2* was suppressed or enhanced, respectively, and a significant number of WNT-target genes were differentially expressed (42).

WNT responses upon encounter of pathogenic and non-pathogenic *Escherichia (E.) coli* have been investigated to some extent in mouse models *in vivo*. Mono-colonization of mice with *E. coli* F18 enhanced expression of *Wnt2* in the intestine compared to germ-free mice (50). Bladder infection with uropathogenic *E. coli* (UPEC) induced rapid downregulation of *Wnt5a* expression in the urothelium of infected mice, which was partially attributed to the bacterial virulence and adhesion factor, FimH (58). This observation seems to contrast a small increase of *WNT5A* expression described in a human urothelial cell line infected with UPEC *in vitro* (59). Yet, exposure of mouse thioglycolate-elicited peritoneal macrophages exhibited a marked decrease in *Wnt5a* mRNA expression when exposed to a non-pathogenic *E. coli* strain, while expression of all other WNT ligands remained unaltered at the time point analyzed (44). Decreased Wnt5a protein and decreased *Ctnnb1* mRNA expression in the mouse macrophage cell line RAW264.7 have also been reported upon infection with *Pseudomonas (P.) aeruginosa* (57) (63).

While several studies reported WNT5A expression to be responsive to macrophage encounter with Gram-negative bacterial pathogens, it remains to be defined whether opposing directions of the regulation of *WNT5A* expression reflect pathogen-specific responses, cell-type-dependent variations, or species-specific differences between humans and mice. Some indication that the latter apect might indeed be of importance comes from studies of macrophages stimulated with lipopolysaccharide (LPS), a major cell wall component of Gram-negative bacteria. Increased *WNT5A* expression has been noted in LPS-stimulated human monocytes, the human monocytic THP-1 cell line, as well as human bronchial epithelial BEAS-2B cells (19, 34, 53, 60, 61). LPS-induced *WNT5A* expression in human macrophages occured downstream of Toll-like receptor signaling and nuclear factor kappa B (NF-κB) activation, and was amplified by inflammatory cytokines such as tumor necrosis factor (TNF) and interferon γ (IFNγ) (19, 34, 60). In contrast, LPS stimulation did not significantly increase the relatively low *Wnt5a* mRNA expression of mouse bone marrow-derived macrophages (40), yet LPS stimulation did enhance *Wnt5a* mRNA expression by mouse RAW264.7 macrophages (37). Nevertheless, the amplitude of the *WNT5A* response might also be governed by the nature of the invading bacterial pathogen as suggested by observations that *Porphyromonas gingivalis* LPS induced *WNT5A* mRNA expression in THP-1 cells to a greater extent compared to *E. coli*-derived LPS (60).

Importantly, however, the LPS-induced WNT response encompasses more than WNT5A. LPS stimulation elevated expression of *WNT3* in human monocyte-derived macrophages (52), and *WNT1* in differentiated human THP-1 cells and murine bone marrow-derived macrophages (40, 48). The latter cells also displayed increased expression of *Wnt2b, Wnt5b, Wnt6*, and *Wnt10b* upon LPS stimulation, whereas expression of *Wnt5a, Wnt10a*, and *Wnt11* remained unchanged, and expression of the remaining *Wnt* genes was below the detection limit (40). Systemic challenge of mice with a sub-lethal dose of LPS *in vivo* induced rapid elevation of *Wnt5b, Wnt10a, Wnt10b, Wnt11, Fzd1*, and *Fzd5* mRNA expression in spleen tissue, accompanied by elevated expression of the WNT target gene *Wisp1*. In contrast, expression of *Wnt6, Fzd7*, and *Fzd8* was decreased, whereas *Wnt3, Wnt4, Wnt5a, Wnt16, Fzd3, Fzd6, Fzd9*, and *Fzd10* were not differentially expressed (39). In an ovine model of fetal intra-amniotic LPS exposure, elevated expression of *Wnt7b*, and reduced mRNA expression of *Wnt1, Wnt2, Wnt4*, and *Ctnnb1* were observed in fetal lung tissue (49).

With patterns beginning to emerge in the WNT response to Gram-negative bacteria (e.g., WNT5A expression by macrophages), more detailed insight is required to delineate the impact of cell type-, species-, and pathogen-specific contributions to the amplitude and composition of infection-associated WNT responses.

#### Gram-Positive Bacteria

WNT responses upon infection with Gram-positive bacteria are just beginning to be explored (Table 1). *Staphylococcus (S.) aureus* infection of *Drosophila melanogaster* led to enhanced expression of *Wnt4* (55). Expression of other WNT ligands was not determined in this study, leaving it to be determined how *S. aureus*, and other pathogens, affect WNT expression in *Drosophila*. *S. aureus* infection of *Caenorhabditis elegans* induced elevated expression of the WNT2 homolog *cwn-2*, and suppressed expression of the WNT1 homolog *mom-2* and the FZD homolog *mom-5* (45). A comprehensive analysis of WNT expression in the Pacific white shrimp *Litopenaeus vannamei* revealed pronounced upregulation of the mRNA expression of multiple WNT ligands, including *LvWnt5, LvWnt6, LvWnt9*, and *LvWntA* in different organs upon *S. aureus* infection (46). Expression of *Wnt5a* and *Fzd4* by murine macrophages marginally increased upon infection with *S. aureus* (44). In contrast, infection of RAW264.7 mouse macrophages with *Streptococcus (S). pneumoniae* has been reported to suppress Wnt5a protein expression (57). Sequencing analyses of lung tissue of mice vaccinated intranasally with *S. pneumoniae* deficient for the autolysis-inducing factor pep27 revealed enhanced expression of *Wnt4, Wnt5b, Wnt7a*, and *Wnt7b*, and impaired *Wnt2b, Wnt3, Wnt6, Wnt9a*, and *Wnt9b* mRNA expression (51). Kinase activity profiling in mouse lung tissue of *S. pneumoniae*-infected mice indicated a reduction in β-catenin-stabilizing signals associated with a decrease in β-catenin protein expression (62). Thus, due to the paucity of information it is currently largely unknown if host cell encounter with pathogenic Gram-positive bacteria directly modulates WNT responses and signaling capabilities.

#### Mycobacteria

Mycobacterial infections induce significant alterations in the expression of WNT signaling components in infected tissues of a variety of host organisms (Table 1). Macrophage-associated *WNT5A* expression was initially described in tuberculosis lung granulomas (34), and *WNT5A* and *FZD4* mRNA expression was significantly elevated in peripheral blood mononuclear cells of tuberculosis patients (44). *Mycobacterium (M.) tuberculosis* infection of C57BL/6 mice enhanced lung mRNA expression of *Wnt1, Wnt6, Wnt10a, Fzd1*, and *Fzd5*, while reducing expression of *Wnt2, Wnt2b, Wnt3a, Wnt4, Wnt5a, Wnt7a, Wnt8a, Wnt10b*, as well as *Fzd3, Fzd7, Fzd8, Fzd9*, and *Fzd10* (40, 41)*. M. marinum* infection of zebrafish enhanced expression of *wnt5a*, yet suppressed expression of multiple other WNT ligands, receptors and WNT pathway regulators (54). Regulation into opposing directions was noted for some WNT network components, depending on the virulence of the infecting *M. marinum* strain (54). Macrophages are major host cells for mycobacteria and have been identified as a significant source of WNT expression during mycobacterial infection. *In vitro* studies showed that infection of monocytes and macrophages of human and mouse origin with mycobacteria across a virulence spectrum (*M. tuberculosis, M. avium, M. bovis* Bacillus Calmette-Guérin) greatly enhanced expression of *WNT5A* (34, 44). Importantly, expression and induction of *WNT5A* in human macrophages was more pronounced compared to mouse cells. In *M. tuberculosis*-infected mice, expression of Wnt6 was localized to macrophages in lung granulomas, and *Wnt6* mRNA expression was significantly elevated in murine bone marrow derived macrophages infected with *M. tuberculosis* or *M. avium* (40). Taken together, the experimental evidence to date suggests that upregulation of WNT5A by mycobacteria-infected macrophages may be evolutionarily conserved between humans, mice and possibly other species. Nevertheless, expression of other WNT ligands by infected macrophages remains to be explored more systematically across species. Moreover, WNT/WNT receptor expression in infected tissues requires cellular context for more detailed understanding of where WNT responses occur upon encounter of pathogenic mycobacteria.

#### Toward Defining Patterns in the Host WNT Response to Bacterial Infections

A WNT response consistently reported for human, and to some extent murine, macrophages to diverse microbial challenges appears to be regulation of *WNT5A* expression. Yet, as it becomes clear that host WNT responses to bacterial infection reach well beyond differential expression of WNT5A, it will be essential to delineate whether patterns of WNT pathway activity are stereotypical responses of distinct host cell types and tissues to microbial insult, and/or how these responses are defined by the nature of the invading pathogen. With increasing insights into WNT responses to infection arises the need to understand WNT responses in human disease. Studies in patients with severe sepsis and septic shock highlight the complex nature of the host WNT response to microbial insult. Comparisons of blood gene expression patterns in patients with septic shock compared to healthy controls, revealed elevated expression of *WNT5B* and *WNT11*, whereas the expression of *WNT1, WNT2B, WNT3, WNT6, WNT7A, WNT9A, WNT10A, WNT10B*, and *WNT16* was significantly reduced (39). Patients with severe sepsis had elevated WNT5A serum levels, and patients with sepsis-associated acute respiratory distress syndrome displayed elevated WNT5A protein expression in lung tissue (19, 68, 69). An increase of WNT5A protein serum concentrations appeared to correlate with disease progression, whereas a decrease was associated with recovery in critically ill sepsis patients (68). However, *WNT5A* mRNA expression in whole blood was very low and not significantly different between healthy controls and septic shock patients, whereas alterations in the expression of other WNT ligands was more readily detectable (39). Whether dynamic changes in the expression of WNT pathway components accompanying severe acute infections can be exploited for the development of easily assessible biomarkers remains to be determined. Signatures that might enable patient stratification or rapidly identify classes of causative bacteria are worth exploring.

### Protozoal and Fungal Infections

WNT responses to infections with protozoa and fungi are less well-explored (Table 2). In mice intraperitoneally inoculated with the protozoan parasite *Trypanosoma (T.) cruzi*, protein expression of Wnt3a, Wnt5a, and β-catenin in splenic mononuclear cells increased with disease progression (74). Similar patterns were observed for Wnt3a and Wnt5a mRNA and protein expression in murine bone marrow-derived macrophages (BMDMs) (74). *In vitro* experiments indicated enhanced expression of *Wnt3a* and *Wnt5a, Fzd4, Fzd6, Fzd8*, and *Fzd9* upon *T. cruzi* infection of murine BMDMs. In contrast, *Leishmania donovani* infection of mouse RAW264.7 macrophages resulted in diminished expression of *Wnt5a*, whereas other WNT ligands and sigaling components were not assessed (80). In human corneas infected with the fungus *Aspergillus (A.) fumigatus, WNT5A* expression was found to be significantly higher than in uninfected corneal tissues. WNT5A mRNA and protein expression were also enhanced by *A. fumigatus* infection of human THP-1 macrophages (78). Murine peritoneal macrophages infected with *Candida albicans, A. fumigatus*, or *A. flavus* or stimulated with the fungal and bacterial cell wall component Curdlan displayed elevated Wnt5a expression (79). More comprehensive profiling of the WNT network will be required to assess the quality of WNT responses by protozoal and fungal infections and determine to what extent WNT expression and signaling are defined by the host cell vs. the nature of the encountered pathogen.

**Table 2:** WNT responses induced by viral, fungal, and protozoal infection in experimental systems.

	**Viruses**	**Fungi**	**Protozoa**
WNT1	↑ mRNA HepC Huh7 (70) unaffected mRNA HIV hPdA (71)	–	–
WNT2	↓ mRNA IVA murine lungs (72) ↑ mRNA HCMV hMSC (73) n.d. mRNA HIV hPdA (71)	–	–
WNT2B	↑ mRNA HIV hPdA (71)	–	–
WNT3	unaffected mRNA HIV hPdA (71)	–	–
WNT3A	↓ mRNA IVA murine lungs (72) n.d mRNA HIV hPdA (71)	–	↑ protein *T. cruzi* mSMC and mBMDM (74) ↑ mRNA *T. cruzi* mBMDM (74)
WNT4	n.d. mRNA HIV hPdA (71)	–	-
WNT5A	↑ mRNA EBV hNC (75) ↓ mRNA HCMV hFF (76) ↑ mRNA HIV mSDH (77) n.d mRNA HIV hPdA (71)	↑ mRNA, protein *A. fumigatus* THP-1 and human corneas (78) ↑ mRNA *C. albicans, A. flavus, A. fumigatus*, Curdlan mPM (79)	↓ mRNA *L. donovani* RAW264.7 (80) ↑ protein *T. cruzi* mSMC, mBMDM (74) ↑ mRNA *T. cruzi* mBMDM (74)
WNT5B	↓ mRNA HCMV hFF (76) unaffected mRNA HIV hPdA (71)	–	–
WNT6	n.d mRNA HIV hPdA (71)	–	–
WNT7A	n.d mRNA HIV hPdA (71)	–	–
WNT7B	n.d mRNA HIV hPdA (71)	–	–
WNT8A	n.d mRNA HIV hPdA (71)	–	–
WNT8B	n.d mRNA HIV hPdA (71)	–	–
WNT9A	unaffected mRNA HIV hPdA (71)	–	–
WNT9B	unaffected mRNA HIV hPdA (71)	–	–
WNT10A	n.d mRNA HIV hPdA (71)	–	–
WNT10B	↓ mRNA IVA murine lungs (72) ↑ mRNA HIV hPdA (71)	–	–
WNT11	n.d mRNA HIV hPdA (71)	–	–
WNT16	↑ mRNA HPV18 HaCat (81) unaffected mRNA HIV hPdA (71)	–	–
Effects not specific to individual WNT proteins	↓*Fzd2, Lrp4, Tcf-3* mRNA IVA murine lungs (72) ↑ WNT/β-catenin signaling Rift Valley fever virus HEK293T (82) ↑β-catenin degradation HCMV hDF and hPET (83) ↑β-catenin protein HepB Huh7 (84) β-catenin stabilization HepC HEK293T (85), EBV hB-cells (86), Vaccinia virus HEK293T (87) ↓ protein β-catenin, no effect on DVL2,3 HCMV hFF (76)	–	↑β-catenin protein *T. cruzi* mSMC and mBMDM (74) ↑*Fzd4, 6, 8, 9* mRNA *T. cruzi* mBMDM (74)

*mRNA and protein expression of WNT signaling molecules in response to infection. ↑, upregulation; ↓, downregulation; –, indicative of no data; n.d., not detectable; protein, protein expression; mRNA, mRNA expression; m, murine; h, human; MSC, mesenchymal stem cells; PdA, primary-derived astrocytes; SMC, spleen mononuclear cells; BMDM, bone marrow-derived macrophages; NC, nasopharyngeal carcinoma; FF, foreskin fibroblasts; SDH, spinal dorsal horn; DF, dermal fibroblasts; PET, placental extravillous trophoblasts*.

### Viral Infections

WNT responses to viral infections have been studied in the context of a limited number of viral infections (Table 2). HIV infection elevated *WNT2B* and *WNT10B* expression by human primary astrocytes (71), whereas expression of *WNT1, WNT3, WNT5B, WNT9A, WNT9B*, and *WNT16* remained unaffected, and *WNT2, WNT3A, WNT4, WNT5A, WNT6, WNT7A, WNT7B, WNT8A, WNT8B, WNT10A*, and *WNT11* expression was below the detection limit of the assay (71). HIV infection of mouse neuronal cells of the spinal dorsal horn elevated *Wnt5a* mRNA expression (77). *WNT5A* expression was also upregulated in Epstein Barr virus (EBV)- infected nasopharyngeal carcinoma epithelial cells (75). Influenza A infection of mice resulted in impaired expression of *Wnt2, Wnt3a, Wnt10b, Fzd2, Lrp4*, and *Tcf3* in infected lung tissues (72). Human cytomegalovirus (HCMV) infection of human foreskin fibroblasts was associated with *WNT5A* and *WNT5B* downregulation (76), whereas HCMV infection elevated *WNT2* expression in human mesenchymal stem cells (73). HCMV infection of dermal fibroblasts, placental extravillous trophoblasts, and foreskin fibroblasts was associated with degradation of β-catenin (83). In contrast, β-catenin stabilization was observed in human B cells infected with EBV (86), vaccinia virus-infected HEK293T cells (87), hepatitis B virus-infected Huh7 cells (84), and hepatitis C virus-infected HEK293T cells (85). These reports indicate responsiveness of the WNT signaling network to viral infections. Modulation of β-catenin stabilization might be indicative of viral exploitation of host cell replication and apoptosis. Yet, the WNT responses associated with viral infection noted thus far show no discernible patterns, likely due to the paucity of comprehensive analyses. Systematic comparisons of host cells and different viral classes are required to assess whether there are WNT network signatures that are indicative of a viral infection.

## WNT Functions in the Host Response to Infection

**Figure 3:**
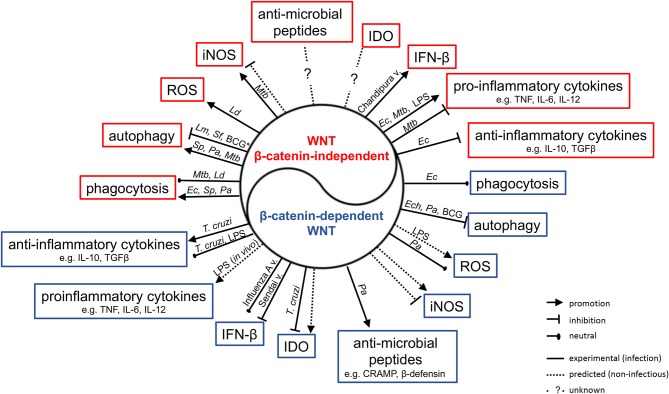
Impact of WNT ligands that activate β-catenin-independent vs. β-catenin-dependent signaling on the functions of macrophages and myeloid DCs in the context of infection with phylogenetically diverse pathogens. *Mtb, Mycobacterium tuberculosis; Ld, Leishmania donovani; Ec, Escherichia coli; Sp, Streptococcus pneumonia; Pa, Pseudomonas aeruginosa; Ech, Ehrlichia chaffeensis; Lm, Listeria monocytogenes; Sf, Shigella flexneri; T. cruzi, Trypanosoma cruzi*; v., virus; *Autophagy in IFNγ-activated macrophages.

The realization that the WNT network is responsive to infections has driven significant interest in delineating its roles in host defense and immune responses. There is increasing evidence that WNT ligands (and other ligands for WNT receptors) contribute to the host control of phylogenetically diverse pathogens in non-vertebrates and vertebrates (57, 74, 80, 88, 89). Some associations between polymorphisms in WNT network genes, and susceptibility and quality of the immune response to infection have been suggested (90–93). Professional antigen-presenting cells (APCs) such as macrophages and dendritic cells have been studied intensively as sources and targets of WNT ligands (19, 40, 44, 94, 95). Roles for WNT ligands in orchestrating phagocytosis, antimicrobial defense and inflammatory cytokine responses have been indicated (Figure 3, Table 3) (48, 98, 100). WNT ligands have also been implicated in the cellular differentiation and functional polarization of APCs and T cells, bridging of innate and adaptive immune responses (34, 94), and shaping lymphocyte functions (107–112).

**Table 3:** WNT functions in shaping host cell defense mechanism.

	**Phagocytosis**	**Autophagy**	**ROS**	**NOS**	**Antimicrobial peptides**	**Inflammatory cytokines**
WNT1	–	–	–	↑ iNOS LPS stimulated THP-1 (48)	–	↑ IL-6 ↑ TNF LPS stimulated THP-1 (48)
WNT2	↑ virus uptake *D. melanogaster* S2 (96)	–	–	–	–	–
WNT2B	-	–	–	–	–	↓ Sendai virus-induced *IFN*β*1* HEK293T (97)
WNT3A	unaffected *E. coli* DH5α RAW264.7 (98)	↓*M. bovis* BCG RAW.264.7 (99)	unaffected *P. aeruginosa* RAW264.7 (100) ↑ NADPH oxidase and hydrogen peroxide HUVEC (101)	-	↑ mRNA β-defensin 1 and CRAMP *P. aeruginosa* RAW264.7 (100)	↓ TNF, IL-6, IL-1β *P. aeruginosa* RAW264.7 (100)
WNT5A	↑*E. coli* DH5α RAW264.7 (98) ↑ latex beads RAW264.7 (80) ↑*S. pneumoniae and P. aeruginosa* RAW264.7 (57) unaffected *L. donovani* RAW264.7 (80) unaffected Mtb hMDM (102)	↑*P. aeruginosa* and *S. pneumoniae* RAW264.7 (57) ↑ *Mtb* infected hMDM (102) ↓*M. bovis* BCG, *S. flexneri, L. monocytogenes*, but not *K. pneumoniae, S. aureus* or *E. coli* (IFNγ-induced autophagy) RAW264.7 (103)	↑NADPH oxidase-mediated ROS production RAW264.7 (80) ↑ ROS ↑ Wnt5a mPM and RAW264.7 (79)	–	–	↑ mycobacteria-induced IL-12 and IFNγ in PPD and mycobacteria-stimulated PBMC (34) ↑ TNF, IL-6, but not IL-10 RAW264.7 ±*E. coli* (98) ↑ TNF, IL-6, IFNβ *E. coli* RAW264.7 (104) and hPBMC (19) ↓ IFNβ Chandipura virus Wnt5a^KD^ RAW264.7 (104) ↑ IL-8, IL-6, IL-1β in hPBMC (19) ↑ IL-10 hMDM, ↑IL-10 promotor RAW264.7 (105) ↑ IL-6, IL-8, IL1β hPBMC (19)
WNT6	–	–	–	–	–	↓ TNF *Mtb* mBMDM (40)
WNT7A	↓ mMDM (106)	–	–	–	–	↓ IL-10, IL-12 ↑IL-6 mMDM (106)
WNT9B	–	–	–	–	–	↓*IFNβ1* Sendai virus HEK293T (97)
WNT11	–	–	–	–	–	↓IL-8 mIEC (56)

*↑, upregulation; ↓, downregulation; –, indicative of no data; protein, protein expression; mRNA, mRNA expression; m, murine; h, human; HUVEC, human umbilical vein endothelial cells; MDM, monocyte-derived macrophages; PBMC, peripheral blood mononuclear cells; BMDM, bone marrow-derived macrophages; IEC, intestinal epithelial cells; MIM, myocardial infarct macrophages; Mtb, Mycobacterium tuberculosis*.

### Considerations for Experimentation

Experimental approaches to deciphering WNT ligand-driven immune functions include utilization of mouse models with genetic deletion of individual WNT ligands or receptors. Use of cell-specific deletion (95, 113) or heterozygous mice (40, 57) is often indicated due to the deleterious impact of global deletion of individual WNT ligands on embryonic development. SiRNA-mediated knock-down of endogenous WNT components (104, 114), interference with WNT/WNT receptor interactions using neutralizing antibodies and recombinant WNT regulators (e.g., sFRPs, DKK) (34, 115), as well as plasmid-based overexpression of WNT ligands, receptors and regulators (63, 116) are commonly utilized, in particular in *in vitro* cell-based studies. Conditioned media from WNT-overexpressing cells and recombinant WNT proteins have also been proven as valuable tools for deciphering WNT functions. Of note, some biological responses of innate immune cells observed upon exposure to recombinant WNT protein preparations have been attributed to Toll-like receptor activation, rather than known WNT receptors (61, 117). The biological importance of this requires further clarification.

As it becomes increasingly evident that multiple WNT ligands are differentially expressed in response to microbial insults, and that WNT ligands are likely to arise from different cellular sources during infection, strategies that broadly target the WNT response as opposed to individual WNT ligands are increasingly employed. Cell-targeted conditional deletion of WLS and PORCN in mouse models, and the use of small molecule inhibitors targeting PORCN activity have proven useful for *in vitro* and *in vivo* studies (39, 42, 80, 98, 112, 118). Similarly, genetic and pharmacologic interference with β-catenin functions as a transcriptional co-activator have been employed to delineate functions of β-catenin-mediated WNT signaling (39, 112, 119). It is important to note, however, that β-catenin stabilization is not exclusively indicative of WNT/WNT receptor engagement, and that microbial ligands and growth factors can induce β-catenin stabilization (120–122). Thus, here we chose to focus mainly on studies that link WNT ligands, their receptors and regulators with host defense to infection.

### Phagocytosis

WNT-induced engagement of the actin cytoskeleton suggests that WNT ligands may play an active role in phagocytosis. Indeed, the *D. melanogaster* glypican dally is a co-receptor in wingless signaling and has been implicated in promoting phagocytosis of a non-pathogenic virus (white spot syndrome virus) by S2 phagocytes. Functional interactions of dally with frizzled 2 and wnt2 in this process were deduced from gene expression analyses (96). In mouse RAW264.7 macrophage-like cells, it has been reported that exposure to Wnt5a-conditioned medium or recombinant Wnt5a enhanced uptake of non-pathogenic *E. coli* DH5α, as well as latex beads. In contrast, Wnt3a-conditioned medium did not enhance phagocytosis (80, 98). Fzd5, as well as Rac-1, PI3K and IKK signaling were implicated in mediating the Wnt5a-driven phagocytic activity. Treatment with liposome-encapsulated Inhibitor of WNT Production-2 (IWP-2), a small-molecule PORCN inhibitor (123), impaired macrophage uptake of *E. coli* DH5α (98). A follow-up study described that exogenously added recombinant Wnt5a also enhanced RAW264.7 cell phagocytosis of *S. pneumoniae* (Gram-positive) and *P. aeruginosa* (Gram-negative) mediated by Rac-1 and Dvl (57). Mice pre-treated with IWP-2 displayed enhanced bacterial burden within peritoneal cells at 2 h post-intraperitoneal infection, and within lung homogenates 5 h after intranasal infection with *P. aeruginosa*. Similarly, more viable *P. aeruginosa* were recovered from peritoneal cell lysates of *Wnt5a*^+/−^ mice compared to *Wnt5a*^+/+^ mice (57). These observations further suggest a role for Wnt5a, and potentially other WNT ligands in host cell uptake of *P. aeruginosa*. However, treatment of RAW264.7 cells with recombinant Wnt5a did not alter internalization of *L. donovani* (80), and siRNA-mediated knock-down of endogenous WNT5A did not impair phagocytosis of *M. tuberculosis* by human monocyte-derived macrophages (102). Thus, the effects of WNT5A on phagocytosis of bacterial pathogens requires further investigation, including comparisons of extracellular alongside intracellular pathogens and macrophages of different origins.

*E. chaffeensis* is an obligate intracellular pathogen that infects mononuclear cells through caveolae-mediated endocytosis and resides in intracellular vesicles that retain characteristics of early endosomes (124). *E. chaffeensis* tandem repeat proteins (TRPs) are secreted effectors of *E. chaffeensis* that have been shown to interact with host cell proteins, including components of the WNT signaling network (125, 126). Phagocytosis of TRP120-coated microspheres by human monocytic THP-1 cells was impaired by small molecules targeting intracellular signaling components that are also part of the WNT signaling network, such as β-catenin/TCF interactions (FH535), CamKII (KN93), and Rac-1 (NSC23766) (42). In contrast, a PORCN inhibitor (IWP-2) did not impair TRP120-microsphere phagocytosis, suggesting that secreted WNT proteins may not have been directly involved in driving this process. In contrast to phagocytic cells, WNT11 over-expression, but not WNT2 overexpression, by human intestinal epithelial HCT116 cells has been suggested to decrease invasion by *Salmonella enterica* Typhimurium (50, 56). The cellular mechanisms facilitating this protection are unknown and it remains to be established how induction of WNT11 expression by *Salmonella* infection might contribute to pathogenesis *in vivo*.

### Autophagy

Several studies have begun to address how WNT ligands might affect the ability of host cells to control pathogenic bacteria. In the case of non-pathogenic *E. coli* DH5α, exogenous addition of Wnt5a enhanced phagocytosis, but did not alter the ability of RAW264.7 macrophages to rapidly kill the engulfed bacteria (98). In contrast, RAW264.7 macrophages exposed to recombinant Wnt5a displayed a more rapid decline in viable intracellular *S. pneumoniae* and *P. aeruginosa* within the first 2–3 h of infection. Wnt5a-induced killing within the first hours of infection was suggested to be mediated by Rac-1 and Dvl. Mechanistically, the authors implicated enhanced autophagy as the mechanism of Wnt5a-induced enhanced control of engulfed *S. pneumoniae* and *P. aeruginosa* (57). While *S. pneumoniae* is targeted by autophagy in non-phagocytic cells (127), the contribution of autophagy in macrophages to controlling this bacterium had not been reported previously. In contrast, the contributions of autophagy to macrophage control of *P. aeruginosa* require further clarification as beneficial effects for the host as well as the bacteria have been suggested (63, 128–131). It is noteworthy, however, that after the sharp initial decline of viable intracellular *S. pneumoniae* and *P. aeruginosa* in Wnt5a-treated RAW264.7 macrophages, from day 1 onwards the intracellular bacterial burden declined more slowly and at a similar rate in both Wnt5a- and control-treated cells (57). Thus, the cellular mechanisms accelerating the initial bacterial killing might be transient, and could be specific to some pathogens as they did not affect macrophage killing of non-pathogenic *E. coli* DH5a (98). With Wnt5a expression reported to be suppressed by *S. pneumoniae* and *P. aeruginosa* infection of macrophages (57), roles of other WNT ligands responsive to infection (e.g., Wnt4, Wnt5b, Wnt7a, Wnt7b) (51) and the net-outcome of WNT signaling in infected cells will need further exploration. Of note, overexpression of β-catenin in RAW264.7 macrophages has been reported to accelerate killing of engulfed *P. aeruginosa*, which was associated with suppression of autophagy (63).

Beneficial or detrimental impact of WNT-autophagy-crosstalk might be defined by a pathogen's ability to exploit intracellular niches for replication and survival. Intracellular bacterial burden in *E. chaffeensis*-infected THP-1 cells was diminished when cells were exposed to IWP-2, as well as the β-catenin/TCF-1 inhibitor FH535, or the CamKII inhibitor KN93. Small interfering RNA-mediated knock-down of WNT pathway components, including *WNT5A, FZD5, FZD9, LRP6, CTNNB1*, and *DVL2* diminished intracellular bacterial burden over 1-2 days of infection, further supporting the notion that intracellular survival of *E. chaffeensis* in this cell line was facilitated by the action of endogenous WNT ligands (42). A subsequent study indicated that DVL signaling suppressed autophagy and phago-lysosomal maturation in *E. chaffeensis*-infected cells (132). WNT pathway activation (e.g., by Wnt5a) upon infection with *M. bovis* BCG has been reported to interfere with IFNγ-induced activation of autophagy in mouse macrophages, a process facilitated by arachidonate lipoxygenase. The same mechanisms have also been implicated for *Shigella flexneri* and *Listeria monocytogenes* infection (103). A recent study suggested that in human monocyte-derived macrophages infected with *M. tuberculosis*, WNT5A contributed to enhancing autophagy resulting in a small decrease in intracellular bacterial burden. In this study, WNT5A-mediated autophagy was suggested as an effector mechanism of IL-36γ (102). However, as *WNT5A* expression in human macrophages is rapidly induced by *M. tuberculosis* infection (34), this mechanism might represent an amplification of the WNT5A response of these cells as indicated for other cytokines such as TNF (19). Exogenous addition of Wnt3a conditioned medium suppressed association of intracellular *M. bovis* BCG with autophagy machinery in RAW264.7 macrophages, which was associated with impaired mRNA expression of autophagy effectors (e.g., *Atg5, Atg7, Atg12, p62*) (99). With evidence for bi-directional regulation between WNT signaling and autophagy (133–135), and the notion that some pathogens might exploit this for their intracellular survival, the functional consequences of this cross-talk for pathogen control is an area for future pursuit.

### Reactive Radicals

Additional cell-intrinsic host defense mechanisms that may be regulated by WNT signaling include the formation of reactive radicals. Treatment of RAW264.7 macrophages with recombinant Wnt5a induced NADPH oxidase-mediated ROS production, which has been suggested to contribute to the macrophage control of *L. donovani* (80). Exogenous addition of recombinant Wnt3a or Wnt3a-conditioned medium to human umbilical vein endothelial cells induced elevated expression of endothelial NADPH oxidase and production of hydrogen peroxide (101), and GSK3β has been implicated as a negative regulator of LPS-induced NADPH-oxidase 1 expression and production of reactive oxygen species production by macrophages (136). These observations could implicate β-catenin-stabilizing WNTs as drivers of ROS production. Yet, treatment of RAW264.7 macrophages with Wnt3a-conditioned medium did not affect ROS production upon *P. aeruginosa* infection (100). Thus, contributions of WNT ligands, in particular endogenously expressed WNTs to ROS production as an anti-microbial defense mechanism require further investigation.

*Wls*-deficiency in BMDMs of *Wls*^fl/fl^-Lyz2-Cre mice has been reported to significantly increase mRNA expression of inducible nitric oxide synthase (iNOS, encoded by *Nos2*) (137), a phenotype also observed in macrophages isolated from myocardial infarct tissue of *cfms-icre Wls*^fl/fl^ mice (138). This may be reflective of suppression of iNOS expression by autocrine/paracrine WNT signaling. The human iNOS promoter has TCF-4 binding sites and *Nos2* expression and nitric oxide production were shown to be positively regulated by β-catenin and TCF-4 (139). These observations suggest that the balance of β-catenin-dependent and -independent WNT signaling could be important for fine-tuning iNOS expression and activity. Whether this bears consequences for pathogen control needs to be investigated. Nevertheless, enhanced iNOS expression by *Wls*-deficient macrophages may indicate compensatory mechanisms associated with the inability to release WNT proteins from producing cells and significant elevation of WNT gene expression observed in these cells (137). However, such alterations in WNT expression may be cell specific as F4/80^+^ liver macrophages of *Wls*^fl/fl^-Lyz2-Cre mice did not show significant differences in *Wnt4* and *Wnt6* expression (118).

With some indication that WNT ligands may determine a cell's ability for production of reactive oxygen and nitrogen species, there is also evidence that ROS and NO produced in response to microbial insult may regulate WNT responses. For example, peritoneal macrophages isolated from *Nos2*^−/−^ mice showed lower induction of *Wnt5a, Fzd4*, and *Lrp5* mRNA expression upon *M. bovis* BCG infection compared to wild type control cells. Treatment with an NO-donor restored *Wnt5a, Fzd4*, and *Lrp5* expression in *Nos*2-deficient macrophages (44), implicating reactive nitrogen species as potentiators of WNT signaling initiation. Dectin-1/Syk-mediated ROS production by murine RAW264.7 macrophages contributed to β-catenin stabilization (79), although how this might intersect with WNT-driven cellular activation remains to be explored.

### Antimicrobial Peptides

Beta-catenin-stabilizing WNT ligands may also play a role in the expression of antimicrobial peptides. A recent study reported that Wnt3a-conditioned medium elevated the *P. aeruginosa*-induced mRNA expression of cathelicidin-related antimicrobial peptide (CRAMP) and β-defensin 1 in RAW264.7 mouse macrophages, which correlated with a small increase in bacterial killing by these cells (100). Stabilization of β-catenin has also been linked to production of the α-defensins cryptdin-1 and cryptdin-6 by murine intestinal crypts (140). In *C. elegans*, it has been shown that expression of the antimicrobial peptide *clec-60* (human homolog RegIIIγ) upon *S. aureus* infection is dependent upon the β-catenin homolog *bar-1* (45). These observations implicate β-catenin in the transcriptional control of a range of antimicrobial peptides. This encourage analyses on the potential roles of infection-responsive endogenous WNTs in the expression of antimicrobial peptides by infected cells.

### Tryptophan Metabolism

Indoleamine 2,3dioxygenase (IDO) catalyzes the first rate-limiting step in the catabolism of the tryptophan for the formation of active metabolites (141). IDO activity is essential for host resistance to some infections where IDO activity limits the pathogen's access to the essential amino acid tryptophan (142, 143). The PORCN inhibitor IWP-L6 and the β-catenin inhibitor iCRT14 enhanced IDO expression and activity in *T. cruzi*-infected murine macrophages, which was associated with enhanced control of intracellular parasites (74). This suggests that endogenous WNT expression and associated β-catenin stabilization in *T. cruzi*-infected macrophages suppressed IDO expression in this context. It will be interesting to explore whether induction of WNT/β-catenin signaling by *T. cruzi* is an active strategy of subverting host defense mechanisms. Importantly, β-catenin activity in CD11c^+^ APCs has been associated with induction of IDO expression and the attainment of a tolerogenic phenotype in DCs (144, 145). Whether these apparent differences are reflective of the cellular context (macrophages vs. CD11c^+^ dendritic cells) or the immune responses (parasite infection vs. sterile inflammation) are worth further investigations.

### Anti-viral State and Type I Interferon Responses

GSK3β activity and β-catenin functions have been implicated in the positive or negative regulation of type I interferon (IFN) responses associated with protection or susceptibility of cells to viral infection (82, 97, 146–152). In some studies, direct contributions of endogenous WNT ligands has been confirmed. For example, siRNA-mediated knock-down of Wnt5a in mouse bone marrow-derived macrophages and RAW264.7 cells impaired Chandipura virus-induced IFNβ production associated with enhanced viral load in infected cell cultures (104). WNT2B and WNT9B were identified as negative regulators of Sendai virus-induced interferon beta (*IFN*β*1*) expression, and inhibition of GSK3β-controlled virus-induced type I IFN responses in a β-catenin-dependent manner in a range of human cell lines and primary cells (97). SiRNA-mediated knock-down experiments in human bronchio-epithelial cells (HBECs) identified WNT5A and DKK1 as positive, and FZD5, DVL3, SFRP5, WNT7B, WNT9B as negative regulators of influenza A PR8 replication (114). Knock-down of *WNT2* and *WNT3* (but not *WNT1, CTNNB1, or LEF1*) impaired infection of HeLa cells by Dengue virus (153). Enhanced control of flaviviruses was associated with enhanced type I IFN signaling via interferon regulatory factor (IRF)-3 activation and interferon response gene expression. It was proposed that this was facilitated by cross-regulation and physical interactions between TANK-binding kinase-1 (TBK-1, which phosphorylates IRF-3) and GSK3β (153). However, examples of β-catenin-stabilizing WNTs not affecting virus-induced interferon responses also exist (154).

### Inflammation

WNT signaling has been ascribed both pro-inflammatory and immune-regulatory properties. The paradigm developed over the past decade or so suggests that WNT ligands triggering β-catenin-independent signaling exert pro-inflammatory functions, whereas WNT ligands driving β-catenin stabilization have anti-inflammatory or immune-modulatory effects. These emerging concepts of WNT ligands orchestrating inflammation and immune cell functions have been reviewed and commented on extensively over time (19, 120, 155–163). Here we have chosen to specifically focus on examples for pro-inflammatory and regulatory effects of endogenous WNT ligands.

It is increasingly recognized that the WNT response upon infection or microbial challenge comprises complex changes across multiple WNT ligands, receptors and regulators (Tables 1, 2). Moreover, WNT receptors exhibit a degree of promiscuity for WNT ligands (164, 165). Thus, the concerted action of WNT ligands and the consequences for local and systemic inflammation in the context of infection require careful consideration. Use of small molecule inhibitors of PORCN (e.g., IWP-2) indicated net pro-inflammatory roles of WNT ligands in mouse models of LPS-induced endotoxemia and *E. coli-*induced bacterial peritonitis (39, 98). Moreover, two studies utilizing small molecule inhibitors of β-catenin functions as transcriptional co-activator (ICG001, iCRT3) independently revealed pro-inflammatory functions of β-catenin in LPS-induced endotoxemia and cecal ligation and puncture (CLP)-induced peritonitis (39, 119). This challenged the current paradigm of anti-inflammatory roles of β-catenin stabilization and urges further studies to understand the contributions of β-catenin in different (immune) cells to inflammatory responses *in vivo*. Moreover, which of the individual WNT ligands responsive to infection are responsible for the pro-inflammatory functions *in vivo*, and what role selective downregulation of regulatory WNTs might play in this context remains to be explored in more detail.

Significant focus by some of the earliest studies has been on WNT5A, a WNT family member implicated in driving pro-inflammatory cytokine responses by myeloid cells via β-catenin-independent signaling (19, 34–36, 166). Endogenous WNT5A has been shown to positively contribute to pro-inflammatory cytokine production by monocytes and macrophages in the context of *Mycobacterium* and *E. coli* infection, as well as LPS stimulation (19, 34, 104). Knockdown of WNT5A in primary human bone marrow stromal cell also impaired basal and LPS-induced release of pro-inflammatory cytokines and chemokines (167). Inhibition of endogenous Wnt5a in a mouse model of HIV-induced neuroinflammation reduced gp120-induced pro-inflammatory cytokine responses *in vivo* (77). However, Wnt5a has also been implicated in impairing dendritic cell functions and creating an immune suppressive environment in a mouse melanoma model. Importantly, this was attributed to Wnt5a mediated β-catenin stabilization (168), which contrasts the pro-inflammatory roles of Wnt5a affected by β-catenin-independent signaling upon microbial challenge. This highlights that the receptor/signaling context rather than the WNT ligand might guide the functional outcome of WNT signaling.

Evidence for net anti-inflammatory functions of WNT ligands can be deduced from enhanced pro-inflammatory cytokine release and decreased release of regulatory TGF-β by *T. cruzi*-infected murine macrophages in the presence of PORCN (IWP-L6) and β-catenin/TCF inhibitors (iCRT14) (74). In this study, it was noted that neither PORCN nor β-catenin inhibitors affected *T. cruzi*-induced IL-10 production by infected macrophages *in vitro* (74). Similar results were observed in an *in vivo* LPS-induced endotoxemia model (39). These observations highlight that IL-10 may not be susceptible to WNT regulation in all contexts.

An example of infection-induced expression of a specific endogenous WNT ligand being associated with suppression of pro-inflammatory cytokine responses comes from *M. tuberculosis*-infected mouse macrophages. Bone marrow-derived macrophages from *Wnt6*-deficient mice displayed elevated TNF expression and secretion upon *M. tuberculosis* infection (40). That immune-suppressive roles of individual WNT ligands could be vital for host survival upon bacterial infection has been demonstrated for WntD in *Drosophila*. WntD-deficiency rendered flies more susceptible to *L. monocytogenes* infection and this was attributed to WntD curbing lethal inflammation by negatively regulating expression of the inflammatory mediator edin via suppression of Dorsal, an NF-κB family member (88). Inhibition of intracellular cell signaling cascades that drive pro-inflammatory cytokine expression (e.g., NF-κB) has been implicated as one of the mechanisms by which β-catenin-stabilizing WNT ligands negatively regulate inflammation (169, 170). Evidence on how this contributes to shaping cellular immune responses and inflammation during infection in complex *in vivo* settings will be invaluable to further affirm this regulatory feedback mechanism.

## Functional Fate of Macrophages and Dendritic Cells With Implications for T-Cell Responses

WNT ligands have been implicated in defining the functional polarization and differentiation of macrophages and dendritic cells. These innate immune cells are critical in shaping inflammation and antimicrobial defense, and in instructing adaptive immune responses in their role as professional antigen presenting cells (APCs).

### Macrophage Polarization

Macrophages exhibit functional plasticity along a multi-dimensional spectrum directed by external and internal stimuli such as microbial products, cytokines, oxygen availability and cellular metabolism (171, 172). Accordingly, phenotypic classification of macrophages based on relative induction or suppression of the transcription of individual genes has limitations. Nevertheless, expression of iNOS is commonly associated with (M1-type) inflammatory macrophages, whereas elevation of arginase 1 (Arg1) expression has been associated with (M2-type) alternatively activated macrophages. Nevertheless, Arg1 activity is also found in M1 macrophages regulating NO production by iNOS (171). *Wls* deletion in resting mouse bone marrow-derived macrophages was accompanied by elevated expression of *Nos2, Tnf, and Il6*, and reduced expression of the M2-associated gene *Mrc1* (macrophage mannose receptor), without affecting *Arg1* expression (137). This suggests that basal *Wls* activity (and by inference the net impact of released WNT ligands) contributed toward M2 polarization of these macrophages. In contrast, several studies indicated that *Arg1* expression is regulated by WNT ligands in macrophages upon pathogen encounter. For example, the PORCN inhibitor IWP-L6, but not the β-catenin inhibitor iCRT14, decreased *Arg1* expression in *T. cruzi*-infected mouse macrophages, yet without impacting production of reactive nitrogen intermediates (74). *Wnt6*-deficient macrophages expressed less *Nos2* and *Arg1* in response to *M. tuberculosis* infection, yet reactive nitrogen production was not impaired relative to wild type controls (40). Exogenous addition of Wnt3a-conditioned medium promoted the expression of *Arg1* in *M. tuberculosis*-infected murine BMDMs (41). sFrp1-overexpression, which was accompanied by impaired β-catenin signaling, led to reduced expression of *Arg1* and macrophage mannose receptor, CD206 (173). Albeit not evident of endogenous WNT ligands contributing to macrophage polarization, it is worth considering that *in vitro* exposure of macrophages to recombinant WNT ligands (including Wnt1, Wnt3a, Wnt5a, Wnt7a) have returned varying results on their ability to elicit phenotypic changes indicative of alternatively activated macrophages or macrophages tolerized against LPS activation (61, 105, 106, 117).

### Dendritic Cell Maturation and Functions

The impact of exogenously added or endogenously released WNT ligands and contributions of β-catenin signaling on the expression of functional surface markers of DCs (e.g., MHC-I and MHC-II, co-stimulatory molecules, PD-L1, PD-L2) and DC endocytic capacity has been analyzed in a number of studies returning varying results (115, 137, 174–182). Such variability is likely governed by the use of cells from different species; differentiation and culture conditions; use of exogenous modulation through recombinant WNTs, conditioned media, WNT regulators vs. perturbation of endogenous WNT ligands and signaling events, for example by using small molecule inhibitors or genetic perturbations. Moreover, the utility of recombinant proteins and the possibility of alternative receptors interacting with WNT ligands requires further validation (61, 95, 117, 183, 184).

Nevertheless, β-catenin activity in myeloid cells has emerged as a rheostat in immune-regulation and tolerance, specifically elucidated in *in vivo* models of autoimmunity, gut mucosal homeostasis and cancer (95, 120, 162, 183–185). Recent studies implicate direct roles for WNT ligands that act via engagement of LRP co-receptors in this regulatory mechanism. Selective deletion of LRP5/6 in CD11c^+^ APCs (which includes DC and macrophage populations in the intestinal mucosa) rendered mice more susceptible to dextran sodium sulfate (DSS)-induced colitis (95, 144). This was associated with elevated expression of pro-inflammatory cytokines (e.g., TNF, IL-6, IL-1β) and reduced expression of anti-inflammatory/regulatory effectors (e.g., IL-10, IDO), and functional bias toward fostering Th1 and Th17 responses at the detriment of T regulatory cells (Tregs). The microbiome has been implicated as a driver of inflammation in mice with LRP5/6-deficient CD11c^+^ APCs with expression of a stabilized form of β-catenin specifically in CD11c^+^ APCs ameliorating disease pathology and pro-inflammatory responses in the DSS colitis model (144). Similar experimental approaches confirmed a regulatory role for β-catenin expression in CD11c^+^ APCs in mouse models of experimental autoimmune encephalitis (EAE), collagen-induced arthritis, and tumorigenesis (94, 183–185). It is interesting to note that the adjuvant utilized in the EAE model contains mycobacterial antigens and that LRP5/6-deficient DCs exhibited reduced pro-inflammatory and enhanced regulatory cytokine responses upon mycobacterial stimulation *in vitro* (94), suggesting that infection-associated WNT responses might direct APC functions in Treg vs. Th1 and Th17 differentiation. In an OVA-expressing tumor model, Wnt1-overexpression by DCs was associated with reduced T cell receptor stimulation, granzyme B secretion and cytotoxicity by CD8^+^ T cells (186), whereas conditional knockout of LRP5/LRP6 in CD11c^+^ cells resulted in an increase in granzyme B production by CD8^+^ T cells (185). Thus, WNT-mediated activation of APCs also bears consequences for subsequent T cell functionality. Of note, there is some evidence indicating that WNT-mediated β-catenin signaling also orchestrates the differentiation of plasmacytoid DCs (187–190), but consequences for pDC functions remain to be explored.

The aforementioned studies support the view that β-catenin-stabilizing WNT signaling engaging LRP5/6 co-receptors can mediate an immune-regulatory profile of DC functions. In contrast, inducible deletion of Wnt5a and one of its receptors, Ror2, rendered mice more resistant to DSS-induced colitis (113). This was accompanied by diminished pro-inflammatory cytokine responses, including IL-12 expression, and selective impairment in the differentiation of IFNγ-producing CD4^+^ T cells, without impact on IL-17- and IL-10-producing CD4^+^ T cells (113). It was implicated that Wnt5a in this context arose from non-hematopoietic cells such as fibroblasts, whereas Ror2 signaling occurred in the hematopoietic compartment including DCs. Nevertheless, cultured Wnt5a-deficient and Ror2-deficient colonic DCs showed impaired pro-inflammatory cytokine profiles upon LPS stimulation including enhanced IL-12 production and increased responsiveness to IFNγ (113). These observations support the notion of pro-inflammatory roles of Wnt5a expressed by myeloid cells. They also align with data indicating that myeloid cell-derived WNT5A, and likely other WNT ligands, bridge innate and adaptive immunity by perpetuating the IL-12-IFNγ axis in T cell and natural killer T (NKT) cell responses (34, 112, 174). Importantly, however, the roles Wnt5a plays in shaping DC functions may be defined by the receptor/signaling output. This is highlighted by findings that melanoma-derived Wnt5a effected a metabolic shift in DCs from glycolysis to oxidative phosphorylation, which was attributed to β-catenin- and PPARγ-mediated cellular activation. This resulted in tolerogenic DCs that promoted IDO activity and regulatory T cell differentiation. Relevance of this mechanism was translated into an *in vivo* melanoma model in mice (145). It will be important to delineate whether factors specific to the pathophysiological context (e.g., immune regulatory molecules, cytokine milieu) explain the apparently opposing outcomes of WNT exposure on DC functions in melanoma vs. inflammatory disorders.

### T Cell Functions During Infection

Genetic deletion of β-catenin in CD11c^+^ cells was associated with only a small increase in the frequency of CD4^+^ T cells, but no significant changes in the frequency of CD8^+^ T cells, TCRγδ^+^ T cells, NKT cells, Tregs, or T follicular helper cells were observed (183, 191). These findings suggest that β-catenin functions in CD11c^+^ myeloid cells define the quality of T cell responses due to the functional capabilities of APCs, rather than by significantly affecting lymphocyte differentiation. Nevertheless, β-catenin and TCF activation play distinct roles in the development, differentiation and function of innate-like and adaptive lymphocytes, and direct contributions of WNT ligands to these processes have been shown (110, 192, 193).

In a mouse model of lymphocytic choriomeningitis virus (LCMV) infection, TCF-1-deficiency had no effect on the expansion and functions (e.g., IFNγ production and cytolysis) of effector CD8^+^ T cells (194, 195), whereas others reported an increase in effector CD8^+^ T cells associated with enhanced IFNγ and TNF expression (196). In contrast to the apparently opposing observations for effector T cells, these studies consistently showed reduced numbers of memory CD8^+^ T cells, reduced IL-2 expression, and impaired expansion of memory cells upon rechallenge (194–196). However, it was suggested that these TCF-1-mediated effects may not be attributable to β-catenin functions, as conditional knockout of β-catenin in mature T cells did not affect memory T cell numbers or functions upon LCMV and *L. monocytogenes* infection (197). Yet, in a transgenic mouse model of constitutively activated β-catenin/TCF-1-signaling, an increased proportion of memory CD8^+^ T cells and increased IFNγ expression during LCMV, vaccinia virus and *L. monocytogenes* infection were reported (198). These studies indicate that TCF-1 is likely required for CD8^+^ T cell memory formation and functions after infection. The role β-catenin might play in this and whether WNT ligands have a direct contribution to these signaling events requires further investigation.

In an *in vitro* system, depletion of WNT1, 2B, 3 and 5B from astrocyte-conditioned medium reduced the differentiation of CD8^+^ T cells toward a CD4^dim^CD8^bright^ T phenotype in cultures of human peripheral blood mononuclear cells. CD4^dim^CD8^bright^ T cells in the central nervous system are thought to be effector memory T cells important in the control of HIV (71). While this study implicated direct involvement of WNT ligands in the formation of this CD8^+^ T cell subset, it remains to be determined whether WNT ligands mediated this differentiation by acting on the CD8^+^ T cells, or indirectly via APCs (e.g., by shaping the cytokine milieu). To our knowledge, there are thus far only very few links between WNT ligands and CD4^+^ T cell functions during infection. In a susceptible mouse model of *Leishmania major* infection, an inhibitor of Dkk1, which should increase WNT/β-catenin signaling, exhibited reduced numbers of CD4^+^ T cells in the draining lymph node, with subsequent reduced IL-4 and IL-10 expression after *ex vivo* stimulation (199). An *in vitro* study utilizing neutralizing antibodies against WNT5A and FZD5 showed impaired antigen-specific IFNγ production by human PBMCs of antigen-experienced donors re-stimulated with *M. tuberculosis* antigen. As human T cells expressed FZD5, it was hypothesized that WNT signaling can facilitate memory T cell activation (34). However, these studies did not demonstrate that these effects were driven directly by WNT signaling in CD4^+^ T cells, nor did they exclude WNT effects on APC functions. Detailed analyses of the WNT receptor and WNT regulator repertoire of different T cell lineages and subsets should guide targeted interventions with WNT signaling events to delineate the roles infection-associated WNT responses play in shaping T cell effector and memory formation and functions.

## Concluding Remarks and Future Perspectives

The WNT signaling network has been firmly established as an evolutionary conserved integral component of host responses to infection. In-depth understanding of how WNT ligands define immune cell functions is beginning to offer mechanistic insights into the contributions of WNT responses to pathogen control and inflammation. Experiments establishing how infection-associated endogenous WNT responses shape immune cell functionality *in vivo* will be key to deciphering WNT functions in shaping complex immune responses. Thus far, macrophages and DCs, as well as T cells have been a major focus of delineating WNT-mediated immune functions. Knowledge of how WNT ligands shape the functions of other immune cells, including neutrophils, mast cells, natural killer cells, natural killer T cells, innate lymphoid cells, B cells, etc. is required to begin to understand the complexity of immune-related WNT responses.

Considering that the WNT signaling outcome is largely decided by the cellular context at the level of receptor engagement (20), functional redundancy of WNT ligands, or lack thereof, in orchestrating cellular responses of functionally diverse cells in complex tissue environments is an important factor. With a clearer understanding of the WNT receptor and WNT regulator repertoire expressed by different immune- and non-immune cells in responses to infection, it will be important to determine if there are species-specific differences in the consequences of WNT exposure of functionally similar cells. This is especially critical when investing in utilizing animal models for understanding human pathology and calls for systematic analyses of WNT responses in infected tissues across different species. Reporter mice for WNT ligand and receptor expression as well as WNT signaling activity (200, 201) will be invaluable for the temporal and spatial documentation of WNT responses in complex *in vivo* settings, including infections. Comparisons with human specimens, wherever possible, will be critical.

While some consistent patterns of WNT responses begin to arise (e.g., WNT5A regulation in human macrophages), it remains largely unclear whether stereotypical WNT responses to infection exist regardless of the invading pathogen, or whether the nature of the pathogen dictates the WNT response. Comparative studies using phylogenetically diverse pathogens covering spectra of virulence and pathogenesis mechanisms will be essential to distinguish stereotypical and selective responses to microbial infection. In depth understanding of the molecular drivers and regulators of WNT ligand and receptor expression during infection will be invaluable in delineating which microbial factors drive WNT responses. Whereas our understanding of WNT responses and functions during viral and bacterial infections is taking shape, WNT contributions to parasitic and fungal infections remain to be explored in more breadth and depth. Knowledge of the investment of pathogens into actively manipulating the WNT signaling network (202–204) will inform our understanding of pathogenesis mechanisms and roles of WNT signaling in the host defense against infection. Such insights will be essential when exploring WNT response patterns as biological indicators supporting diagnosis, prognosis and choices for clinical management of infectious diseases (205).

Due to the central role of WNT signaling in maintaining tissue homeostasis, including epithelial barrier functions, consequences of immune-related WNT responses reach beyond leukocyte functions. Indications that WNT/WNT receptor interactions shape chemokine responses (186) and cellular metabolism (145) deserve particular attention in the context of immune responses to infection and beyond. Aberrant WNT expression and/or WNT signaling underlying carcinogenesis, fibrosis, and osteoporosis has generated considerable interest in pharmacologically targeting the WNT signaling network (206–209). Understanding the functional nature and temporal regulation of WNT responses in the host response to infection, and other immune settings, is essential for identifying therapeutic opportunities, but also potential risks of pharmacologically targeting the WNT signaling network.

## Author Contributions

JL, JK, and AB wrote the manuscript. TT contributed sections to the manuscript. JL and AB conceived and designed figures and tables. All authors read and approved the final version of the manuscript.

We apologize to colleagues whose primary research work was not integrated into this review due to space constrains or whose contributions to this immense and rapidly growing field was inadvertently overlooked.

### Conflict of Interest

The authors declare that the research was conducted in the absence of any commercial or financial relationships that could be construed as a potential conflict of interest.
